# The Development of Open Access Journal Publishing from 1993 to 2009

**DOI:** 10.1371/journal.pone.0020961

**Published:** 2011-06-13

**Authors:** Mikael Laakso, Patrik Welling, Helena Bukvova, Linus Nyman, Bo-Christer Björk, Turid Hedlund

**Affiliations:** 1 HANKEN School of Economics, Helsinki, Finland; 2 Technische Universität Dresden, Dresden, Germany; Universidade de Brasília, Brazil

## Abstract

Open Access (OA) is a model for publishing scholarly peer reviewed journals, made possible by the Internet. The full text of OA journals and articles can be freely read, as the publishing is funded through means other than subscriptions. Empirical research concerning the quantitative development of OA publishing has so far consisted of scattered individual studies providing brief snapshots, using varying methods and data sources. This study adopts a systematic method for studying the development of OA journals from their beginnings in the early 1990s until 2009. Because no comprehensive index of OA articles exists, systematic manual data collection from journal web sites was conducted based on journal-level data extracted from the Directory of Open Access Journals (DOAJ). Due to the high number of journals registered in the DOAJ, almost 5000 at the time of the study, stratified random sampling was used. A separate sample of verified early pioneer OA journals was also studied. The results show a very rapid growth of OA publishing during the period 1993–2009. During the last year an estimated 191 000 articles were published in 4769 journals. Since the year 2000, the average annual growth rate has been 18% for the number of journals and 30% for the number of articles. This can be contrasted to the reported 3,5% yearly volume increase in journal publishing in general. In 2009 the share of articles in OA journals, of all peer reviewed journal articles, reached 7,7%. Overall, the results document a rapid growth in OA journal publishing over the last fifteen years. Based on the sampling results and qualitative data a division into three distinct periods is suggested: The Pioneering years (1993–1999), the Innovation years (2000–2004), and the Consolidation years (2005–2009).

## Introduction

### Background

Like many other industries involved in content delivery, scientific publishing has seen new challenges and opportunities with the wide adoption of the Internet. In the early days of the Web, before the 1990s, electronic mailing lists were a popular method for distributing longer strings of text, like journal articles, to groups of people. Since then, technology and web standards have rapidly progressed and matured. Journal articles are now with increasing frequency being both offered and retrieved in digital formats online rather than through physical, printed volumes. Most well-established journals have added digital publishing as a complementary service to their paper editions.

Now that the Internet has enabled low-cost distribution of digital content, the access restrictions put in place to protect and monetize said content have been a topic for active discussion, not least with regard to research results produced with public funding [1], [2], [3]. The costs involved in providing an online-only journal are noticeably different from those of printing and shipping physical journal volumes, with the major online-only cost posts being copy-editing, web hosting, and the maintenance of a functioning mechanism for peer-review. For a comprehensive review of economic implications of alternative publishing models see [4]. When paper issues were the only available option, a wide enough subscriber base was a condition for sustainability of a journal. Open Access business models have been introduced in parallel to traditional subscription-based models; a journal might charge authors for submissions or rely on advertising revenue as a source of income. Additionally, Open Access journals are not only unique because of their paperless operation, but because they offer new possibilities for niche- and emerging subject areas to establish dedicated research outlets.

In summary digital content delivery has, within a relatively short time-span, shifted the landscape of scientific publishing considerably and opened up the market for alternative ways of distributing scientific literature. At the same time as the process of finding, acquiring, and consuming scholarly content has been revolutionized by technology, the access restrictions to scientific literature have been scrutinized with different arguments and perspectives. Open Access is a new technology-enabled business model, which is gaining increasing acceptance.

### Definitions

Open Access (OA), in the context of scholarly publishing, is a term widely used to refer to unrestricted online access to articles published in scholarly journals. Previous research has identified two distinct ways of obtaining open accessibility to scientific research results: Gold OA and Green OA.

Gold Open Access is a form of OA where the document is made available by the publisher to whom the document has been submitted. It has been suggested that 8.5% of all scholarly journal volume for 2008 is available through some form of Gold OA [5]. Gold OA means that the content of the actual journal publishing the article is, either totally or to some extent, freely accessible to the public. The Gold OA category covers a diverse spectrum of publications, including everything from small journals publishing a handful of articles annually to big journals publishing hundreds of articles in the same time frame. The Gold OA category can be further subdivided based on the degree or extent of journal content availability. Direct OA is when the whole journal is published Open Access without any limitations, and is estimated to account for 62% of all Gold OA [5]. Other journals keep the most recent content accessible only to paying subscribers, but as time passes, the embargo is lifted and the content is made available to all. This variant is called Delayed OA, and constituted 14% of all Gold OA [5]. Sometimes an author or the author's institution can pay for an article to be made freely available in an otherwise subscription-based journal. This is referred to as Hybrid OA, and made up 24% of all Gold OA [5].

Green Open Access means self-archiving of the author's work; be it a manuscript, a pre-print version of a manuscript accepted to be published in a scientific journal, or the actual published paper itself. An estimate is that 11,9% of all scholarly articles published in 2008 were available through some form of Green OA [5]. Self-archiving by the author can be done by uploading the paper to the author's personal homepage or to the author's institutional repository. There is also a third major channel for Green OA: subject-based repositories. Subject repositories allow self-archiving of articles which belong to some specific field of science. Good examples of such repositories are ArXiv, which started with physics but has since expanded its scope to cover a variety of research topics, and PubMedCentral for biomedical and life sciences research. Journal articles can be searched for and accessed directly through various aggregated indexes, such as those of popular web search engines, rather than through the publisher's web page or journal homepage. Advanced free-to-use indexing services, such as Google Scholar, which list all available versions of an article against a single bibliometric metadata record, have made it easier than ever before to find full-text versions of articles, even in cases where they are not made available directly by the publisher. Having equal visibility to both publisher-provided copies and copies uploaded either to repositories or other web sites is a completely new dynamic in the traditionally dyadic relationship between the journal and its potential reader.

This study focuses on the most basic form of Gold OA: the Direct OA. For the sake of clarity, from this point on the term ‘OA journal’ is used to refer to scholarly, peer reviewed journals in which all content is available freely on the web from day one, either exclusively online or parallel with a subscription print version, and which can be accessed by anyone with Internet access.

### Aim of the study

The main aim of this study is to produce a reliable and comprehensive analysis of the historical development of OA journals. The analysis covers a time period starting from what can be considered the beginning of the phenomenon in the early 1990s and stretches to 2009. Emphasis has been placed on the use of systematic methods to sample and represent the population in order to construct a reproducible foundation which can be reused should the study be extended upon in the future.

The remainder of the article is structured as follows: first, relevant previous research is reviewed; then, the methods and data collection are presented; finally, the results are detailed, with a discussion interpreting them in a larger context.

### Previous research

A major challenge for research aiming to establish reliable quantitative measurements of OA prevalence and publication volume has been the lack of comprehensive indexing for both OA journals and their articles. Though the situation has improved considerably over the past decade, the lack of article-level indexing for the majority of OA journals still poses a challenge for researchers. The fact that aggregated metrics are not readily available has been a major consideration in, and motivation for, the labor-intensive research design of this study. Previous studies have dealt with this lack of data through a variety of different data sources and collection methods. This section summarizes relevant earlier studies and their major findings, placing the study in a line of existing scientific contributions.

Ware and Mabe [6] measured that the total number of scholarly journals has increased at a steady rate of about 3.5% annually over the last three centuries, while growth in the total number of articles during the same time period has increased at the slightly slower pace of about 3% annually. This trend is important to keep in mind when studying developments in the proportion of OA journals and articles as the growth of OA publications must be compared relatively to the total article volume increase.

Crawford [7] is among the earliest studies documenting the behavior of pioneer OA journals. The study, conducted in 2001, attempted to chart the OA landscape back in 1995. Using data from The Association of Research Libraries, the study found evidence of the existence of 86 journals publishing in 1995 which fulfilled the criteria of free, refereed, and scholarly. Interested in the viability of this novel type of publishing, Crawford also investigated the status and activity of these 86 journals six years later (in 2001). The main finding was that only 49 journals, or 57%, were still actively publishing. There appeared to be a pattern among the majority of the ceased journals, which the author coined ‘the arc of enthusiasm’, where a journal does well during years 2–5, but does not increase the publication volume from the two initial years, only to end up totally inactive or publishing only one or two articles per year after that. Among those that had survived, two distinct groups were discernible: ‘small successes’ (n = 21) which published a steady stream of fewer than ten articles annually, and ‘strong survivors’ (n = 28) which consisted of bigger journals publishing over ten articles annually, with some journals regularly publishing over one hundred articles per year. Considering the speed with which changes happen on the Internet, attempting to measure or reconstruct the open availability of journal articles prior to around 1998 is a challenging task. Fortunately, Crawford conducted both a comprehensive review of OA journal developments between 1995 and 2001, as well as included all journal titles and their annual volumes as part of the article itself.

Another early OA study was carried out by Wells [8], who compiled a list of scholarly OA journals by combining data from several e-journal lists, verifying found journals by visiting their websites. The end result, based on information collected in 1998, was a list of 387 journals, publishing an average of 18 articles per year. While Wells' list was compiled with some limiting criteria – e.g. the list excluded journals lacking any English language on their web pages and did not evaluate the rigorousness of peer review practices among the journals – the list can still be considered an important snapshot of OA publication activity among pioneer journals in their early years. Wells's list was later picked up by Gustafsson [9] who revisited those same journals to check their continued activity and to expand upon the OA journal list with new entries found on the Ulrichsweb Periodicals Database [10], ending up with a total of 317 journals. Around half of the journals Wells [8] originally documented had become inactive, with only 193 of them still publishing actively. This result is in line with the mortality rate noted by Crawford [7]. Interested in finding out more, Hedlund et al. [11] sent out a web survey to the editors of each of the journals in Gustafsson's updated list of 317 OA journals for which contact details were available, 300 in total, and received 60 responses, a response rate of 20%. They found out that during the year 2002, these journals published on average 20 articles each.

The most comprehensive study to date in terms of sample size, and the study with the methodology most similar to this study, is Morris [12]. The study analyzed the results of a labor-intensive data collection process where volunteers manually went through journal websites collecting publication metrics from 1213 of the total of 1443 OA journals listed in Directory of Open Access Journals (DOAJ) [13] at the time. They collected data for the start year of the journal, the year of the most recent published article, and the total amount of articles available. One of the key results of the study was that, on average, 42 articles were published annually per journal. As the author herself notes, the study did not separate between journals which had been born OA and those that had later converted to OA, and focused on the article volume without regard for retrospective archival or conversions from subscription-based to open [12]. So while the data is comprehensive, the results are not suited to represent the availability of OA article volume retrospectively for a given year.

Björk, Roos and Lauri [14] sampled 100 of 1485 OA journals identified by using the Ulrichsweb Periodicals Database. Each journal homepage was visited manually and the publication volume for 2006 was noted. That year, these 100 OA journals were found to publish on average 34,6 articles. However, it should be noted that this study is not directly comparable to studies based on DOAJ sampling as the study excluded titles from large publishers which were known to charge author fees.

In a report covering the first phase of the SOAP (Study of Open Access Publishing) project, Dallmeier-Thiessen et al. [15] analyzed data for all active English language journals listed in the DOAJ, a total of 2838 journals at the time of the study in 2009. The study focused on establishing the current state of open access publishing, analyzing different types of publishers and business models. For journals for which indexed bibliometric data was available the authors used existing external sources, but for a large portion of the population data was gathered by manually visiting the journal websites. The analysis found that the average journal published 43 articles in its most recent active year, which was either 2007 or 2008. Due to its focus on the current status of OA and an extensive publisher-level analysis, the study did not attempt to separate between converted subscription journals and born OA journals.

Some studies have focused on particular predefined segments of OA journals. One recent such study is Edgar and Willinsky [16], who surveyed journals which use the popular Open Journal Systems (OJS) publishing platform. Their study was based on 998 online survey responses (2748 questionnaires sent out, a 36% response rate) from journal editors and managers. While the OJS publishing system was a common denominator, there was considerable variety on several other dimensions. The majority of the journals were founded as OA journals directly on the platform, but there were also many journals which had migrated to the OJS platform either from print-only or from other means of electronic publishing. Only a small number, about 7%, had uploaded back-issues to their archives. The self-reported annual average number of published articles among the responding journals for 2008 was 31.

Within the literature there are also studies which have put particular focus on the subset of OA journals indexed by ISI Web of Science [17], either studying such journals in isolation or in comparisons between indexed and non-indexed journals. Sotudeh and Horri [18] studied the longitudinal evolution of ISI indexed OA journals, with particular emphasis on changes in journal access and access policies. As the result of a thorough retrospective analysis of the journals through various direct and secondary sources, the authors suggested that the ISI OA journals are a relatively heterogeneous lot when it comes to OA evolution, with retractions of OA publishing and experimentation with hybrid OA publishing models being observed. What has been established so far in comparison studies is that indexed journals on average tend to be considerably larger than OA journals. A study based on data from 2003 found 239 OA journals in the ISI index, each publishing on average 92 articles [19], a figure which is considerably higher than the average publishing volume for all OA journals. The study also established that the majority of ISI indexed journals are from within the subject categories of medicine, life sciences, chemistry, and physics, engineering & mathematics.

While the average annual amount of articles per journal is unsuitable as a metric for mapping OA growth directly, such observations contribute towards an understanding of some of the changes that have happened within the population of OA journals over time. Based on the review of existing research it can be concluded that there is a gap in the empirical research regarding the longitudinal development of OA publishing. Most studies on the subject provide only snapshots of a single point in time without including a retrospective analysis founded on the same assumptions as the main study. Studies have shown that both the population of OA publishers and OA journals is heterogeneous, with substantial differences in size. Consequently, generalizations extending between subject areas and years as well as comparisons with the total available mass of academic literature published each year becomes highly unreliable.

## Materials and Methods

### General method description

The study was conducted as a quantitative analysis of the yearly publication volumes of OA journals. The DOAJ, being an actively maintained and well-established index with clear inclusion criteria was used to define the population of peer reviewed scientific OA journals. Since the DOAJ itself only indexes a fraction of the journals on article level, the annual journal volumes had to be obtained from elsewhere. Three options for identifying and collecting this data were considered: Usage of data directly from the ISI and Scopus [20] indexes, a web survey directed to journal editors, and manual data collection from journal web sites. Direct use of ISI and Scopus data is problematic, because only a limited number of the included OA journals are indexed on an article level. Of all the journals listed in DOAJ, only under one tenth have usable information available in ISI, and about one fourth are found to be indexed in Scopus. Furthermore, as both ISI and Scopus typically index large, well-established journals, the sole use of these indexes would also cause considerable bias. Data collected through a survey (e.g. [21]) would have had limitations both due to potentially low response rates leading to a lack of data, but also to bias as the survey rejection pattern is unknown. Obtaining the contact details for the respondents is also potentially problematic considering that many of the journal web-pages have not been updated in years, and e-mail addresses are subject to change. Considering the weaknesses of the aforementioned data collection methods, only a manual data collection process, although labor-intensive, was deemed fit. This way data could be collected in a standardized way for the exact sample of journals desired, thus allowing for the use of regular quantitative methods.

### Sampling

The target population for the study consists of all of the OA journals that have been active at some point during the years 1993 through 2009. We used the selection criteria of the DOAJ regarding coverage, access, and quality to operationalize the definition of the target population. The exact composition of the population based on these criteria is, however, unknown. Some authors in previous studies (e.g. [7], [8]) have individually compiled lists of then existing OA journals. Due to the exponential growth of the number of OA journals, later studies (e.g. [12]) use existing indexes. For the purpose of this study, it is assumed that the DOAJ is a suitable operationalization of the target population and the DOAJ journal index is used as the sampling frame.

As the sample is based on DOAJ data it is important to be aware of the inclusion policies of the service as they have a direct influence on the OA representation and results of the study. Of paramount importance for a retrospective study are aspects that potentially skew faithful representation of past OA volume: 1) The possibility of including ceased journals to the service retrospectively regardless of whether they were OA at time of publication or not, and 2) whether already included journals are cleaned up if they cease to exist. As such information is not publicly available on the DOAJ website, it was confirmed through personal communication with a DOAJ representative that the service does not accept non-active journals for inclusion in its directory, and that ceased journals which websites have become inactive, or otherwise do not fulfill basic DOAJ inclusion criteria, are removed from the directory without any explicit trace of their existence. While these are important aspects to keep in mind when interpreting the results, the DOAJ is still the most comprehensive and detailed index of OA journals available today.

The sampled population contained the 5175 DOAJ journals that were started prior to 2010 as listed in the index at the time this study was initiated in the fall of 2010. Of particular interest concerning this study are both heterogeneity regarding the journal publication volumes and the length of time during which the journals have been providing open access content. The population was analyzed using ISI and Scopus data. It was noted that the journal size distribution was skewed in favor of the big journals, which account for a large portion of the overall article output though they represent only a minority of all the journals present in the population. Another aspect that had to be taken into consideration was that only a small number of OA journals had been active before the year 2000. Relying on random sampling of the whole population with equal probability throughout would provide low precision for estimates of the OA development for the pre-2000 period [22], and the journal size distribution would be so skewed as to cause imprecision in estimating the publication volumes.

As a result of the aforementioned population characteristics, stratified random sampling with unequal probabilities [23] was adopted as the sampling strategy. The population was stratified into two groups: ‘large’ and ‘small and midsized’. The chosen stratification metric was the publishing volume as stated in the 2008 edition of the SCImago Journal & Country Rank Portal [24], and the 2009 data of ISI Web of Science citation database, respectively.

Journals listed in the DOAJ, and also indexed by either Scopus or ISI, and which have published more than 200 articles annually according to the latter indexes, were classified as ‘large journals’. Journals showing lower publication volumes, or those that are absent from the index, were classified as ‘small and midsized’ journals. This classification was based on the rationale that journals with large publication volumes have a high probability of being indexed by either ISI or Scopus. Random samples were selected from both strata, however with different inclusion probabilities as shown in Table 1. For the small and midsized journals, 519 journals were randomly selected from the total stratum. The big-journal stratum, containing 44 journals, was sampled in full.

**Table 1:** Sampling metrics.

Stratum *h*	Description	Stratum size *N_h_*	Fraction of population *N_h_/N*	Sampled units *n_h_*	Selection probability *n_h_/N_h_*	Sampling weight *N_h_/n_h_*
1	small and mid sized	5131	0.991	519	0.101	9.886
2	large	44	0.008	44	1	1
(treated separately)	pioneer OA sample	-	-	304	-	-

The heterogeneity of the journal start years and of the duration of open access to journal content could not be addressed by using stratification. No source of journal-start-year data was found to be reliable enough, and in the absence of a suitable indicator for stratification by start year we chose to collect a separate sample of OA journals known to have been active before the year 2000. This additional pioneer OA sample was taken from the studies conducted by Wells [8] and Hedlund et al. [11], consisting of 304 OA journals publishing in the English language, which were known to be active even before the DOAJ was founded. However, this sample does not form a subset of the sampled population of the OA journals in the DOAJ. To avoid bias, this purposive sample is not combined with the stratified random sample. Comparisons of the data collected from these two separate samples took place only on an analytical level.

### Data collection

Due to the nature of the data to be collected, primarily the annual article volume per journal, the process could be split up and distributed among a team of researchers. However, a standardized way of conducting the work was essential to ensure standardized data entry among several individuals. A software tool was programmed to facilitate the empirical data collection process. The tool uses data from the DOAJ database as a starting point, and allows for non-destructive data manipulation as well as for the entering of entirely new fields of data, such as the yearly amounts of published articles for the journals covered in the sample.

**Figure 1:**
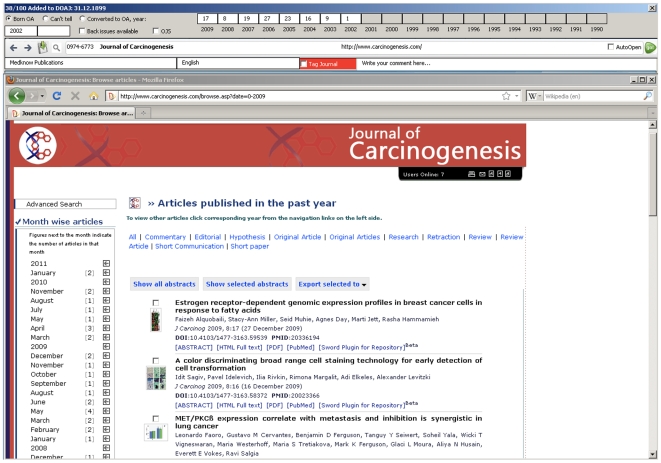
Screenshot of the data collection tool in use.

The use of a specialized data collection tool was considered necessary to facilitate a high standard of collected data, as well as to ensure uniformity in the data produced by the research team members. A screenshot of the tool in use is shown in Figure 1.

Insight into the validity of the journal metadata in the DOAJ was also gained as part of the process: the start year for the journals had to be corrected in 147 cases of the 521 manually sampled journals. Often the DOAJ-supplied start year referred to the year the journal had converted to OA instead of the actual founding year when the first volume was published, as was also pointed out by [18].

### Data analysis

The collected sample data was used to estimate the development of the sampled population. Therefore, the analysis was focused on descriptive statistics. For each year between 1993 and 2009 the total number of publishing journals was calculated, as well as the total number of published OA articles and the average number of published articles per journal per year. Due to the use of a stratified sample, the results were weighted according to the following formula:
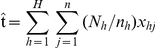
where 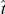 is the estimated value, 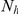 is the total size of stratum 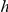, 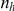 is total number of units sampled from stratum 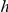, and 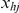 is the value of unit 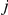 in stratum 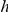.

### Validity and reliability

Throughout the study steps have been taken to ensure the quality of the results with regard to validity, reliability and generalizability. Despite the quantitative character of the study, a holistic understanding of validity as a relationship between the research aims and the empirical setting was emphasized. Thus particular attention has been paid to the operationalization of the research questions. When selecting a suitable population, several available journal indexes were considered and analyzed, and existing studies on Open Access have been taken into account. The population is operationalized using the DOAJ index while at the same time noting its inaccuracies and errors. Furthermore, a stratified sampling was selected to avoid potential bias caused by the heterogeneity of the population, and combined further with a purposive sample based on previous studies. With regard to reliability, a highly structured procedure for data collection was chosen, standardizing not only the data of interest but also the manner in which it was collected. In this manner, the data collection could be distributed among several individuals, making it independent of the judgment of a single person. Throughout the whole process of data collection, the individuals involved met regularly in order to discuss the procedure as well as any problems or unclear cases.

## Results

**Figure 2:**
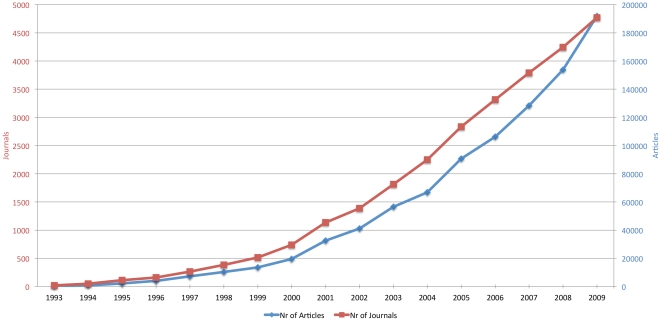
The development of open access publishing 1993–2009.

**Figure 3:**
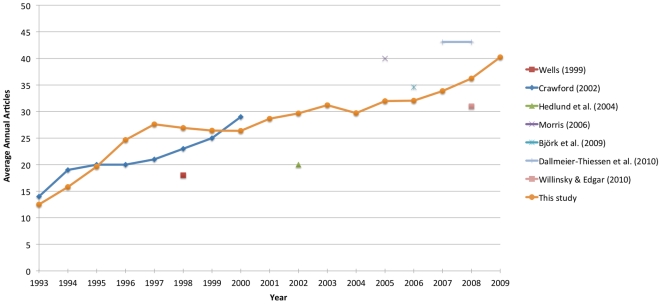
The development of article volume within OA journals.

Figure 2 shows the development of the number of active OA journals and the number of research articles published in them during the period 1993–2009. Table 2 contains the results datasheet on which Figure 3 is based on together with additional measurements and results.

**Table 2:** Results datasheet.

	1993	1994	1995	1996	1997	1998	1999	2000	2001	2002	2003	2004	2005	2006	2007	2008	2009
Nr of Articles	247	781	2174	3972	7285	10 357	13 614	19 521	32 519	41 152	56 619	66 859	90 720	106 245	128 363	153 814	191 851
% Increase to Previous Year		216.0%	178.4%	82.7%	83.4%	42.2%	31.5%	43.4%	66.6%	26.6%	37.6%	18.1%	35.7%	17.1%	20.8%	19.8%	24.7%
Number of observations	2	5	13	19	33	47	62	92	140	170	215	260	322	374	422	469	522
Variance	1615	1609	1597	1601	1586	1571	1567	1579	1599	1697	1852	1674	2086	2083	3105	7900	12459
Standard Deviation	40.18	40.11	39.97	40.01	39.82	39.64	39.58	39.74	39.98	41.19	43.04	40.92	45.68	45.64	55.72	88.88	111.62
Confidence Interval for Total Article Count	5667	5657	5636	5643	5616	5589	5583	5605	5639	5809	6070	5771	6442	6436	7858	12534	15742
Nr of Journals	20	49	111	161	264	385	515	741	1135	1387	1815	2251	2837	3315	3790	4246	4767
% Increase to Previous Year		60.0%	55.4%	31.3%	38.0%	31.4%	25.3%	30.4%	34.8%	18.2%	23.5%	19.4%	20.7%	14.4%	12.5%	10.7%	11.0%
Annual Average of Articles per Journal	12.5	15.8	19.6	24.6	27.6	26.9	26.4	26.4	28.6	29.7	31.2	29.7	32.0	32.1	33.9	36.2	40.2
Confidence Interval For Annual Average	1.10	1.09	1.09	1.09	1.09	1.08	1.08	1.08	1.09	1.12	1.17	1.12	1.24	1.24	1.52	2.42	3.04

The results suggest that, measured both by the number of journals as well as by the increases in total article output, direct Gold OA journal publishing has seen rapid growth particularly between 2000 and 2009. In 2000 we estimate that there were around 19 500 articles published OA, while the number for 2009 is 191 850 articles. The journal count for the year 2000 is estimated to have been 740, and 4769 for 2009; numbers which show considerable growth, albeit at a more moderate pace than the article-level growth. These findings support the notion that OA journals have both increased in numbers as well as increased their average annual output over time.

The average annual publication volume per journal over the period 1993–2009 is shown in Figure 3, together with the results of the previous studies discussed earlier in the article. The data from Crawford [7] were calculated based on the raw numbers appended to the original article. Existing findings suggested an increase in the average number of articles per journal, growing gradually from below 20 in the early 1990s to around 40 in 2009; a notion which is further strengthened by the results of this study.

Descriptive statistics for the samples are shown in Table 3. Two thirds of the pioneer OA sample was OA from the start, as were 60% of the small and mid-sized journals. But among the big journals a small majority (60%) were subscription journals that had converted to OA. A small number of small and mid-size as well as pioneer journals were found to be of questionable scholarly quality. Examples of such include websites where anyone can upload a manuscript and the peer-review is handled by other website visitors commenting on the documents, or journals which consist only of editorials or news items. The output of these journals was not counted and they were excluded from any further analysis. 8% of the pioneer journals had vanished from the Internet and traces of their output could not be found.

**Table 3:** Descriptive statistics.

	Born OA	Converted OA	Conversion year indeterminable	Freely accessible but requires registration	Vanished(website not found)	Converted to paid access	Questionable scholarly quality	Total
Small and mid-size journals (n = 521)	59.88%	35.51%	0.19%	0.77%	0.96%	0.58%	2.11%	100%
Large journals (n = 44)	43.18%	56.82%	0%	0%	0%	0%	0%	100%
Pioneer OA sample (n = 303)	66.01%	17.49%	0%	0.33%	8.25%	4.62%	3.30%	100%

Among the pioneer OA sample there were a handful of journals which had recently switched from being fully OA to a subscription-based model. This phenomenon was interesting to discover as OA journals becoming inactive and losing momentum for various reasons has almost completely dominated the discussion about threats to OA journals. Furthermore, the transition from a subscription-based model to OA has largely been perceived as a one-way process, but this evidence of journals which have either done the reverse or even gone full-circle around speaks for a more complex dynamic. It would be interesting to investigate cases where reverse changes in business-models have happened and study the experiences these journals have had with different models to elaborate further on facilitating factors.

The pioneer OA sample was also used to gain insight into journal mortality since it can be verified from existing research that that particular list of OA journals existed in 2002 [9] and the list has not been modified since, something which cannot be assumed about the records in the DOAJ. Of the 175 Born OA journals which published at least one article in 2000, only 126 were still active in 2009, suggesting a drop of 28% during those nine years. Of the 43 Converted OA journals, 36 were still publishing in 2009, suggesting a 16% drop. The figures are lower than the 43% mortality rate for the period 1995–2001 in the material by Crawford [7]. The difference between the two results can probably be largely attributed to the different time periods they study. The level of establishment and maturity of the studied journals, maturity of technologies and standards related to electronic publishing, and more available knowledge about the conditions of success for online-only journals are likely to be some of the most central influences to the differing results. The attitudes among scholars to publishing in OA journals have also changed over the years.

Of the journals in the small and mid-sized category 13% used the Open Journal System software and another 14% were based on national or regional journal portals (Scielo [25], Redalyc [26] or J-Stage [27]). A large proportion of the journals in the pioneer OA sample still reside on their original websites, of which many appear to be fairly outdated with regards to currently available web technologies and standards. Many are simple static HTML pages linked together without any publishing platform providing back-end automation. Reliable article-level indexing requires journals to provide meta-data in standardized metadata formats, something which in practice necessitates the use of a publishing platform.

### Share of direct Gold OA articles of all scientific articles

After the presentation of the absolute numbers of full OA articles, a natural question to ask is what proportion these constitute of the overall article volume. Since reliable studies of the overall number of scientific peer reviewed articles are scarce it is difficult to find numbers for the denominator of the equation determining the share.

The global number can be estimated using a number of techniques. The starting point can either be journals and articles indexed in the Web of Science (ISI), Scopus, or Ulrich's Periodicals Database. The last of these options is still not complete as there are a large number of peer reviewed journals not listed in Ulrich's, especially journals published in languages other than English.

The earliest reliable estimate of the share of OA articles is a study of the ISI's coverage of Open Access articles done by McVeigh [20]. Using her published data the share of OA articles in ISI could be calculated to 2,9% for 2003. In an earlier study of the 2006 article production [14], the total number of articles published in Ulrich's journals was estimated to be approximately 1 346 000, using a method pioneered by Mabe [28]. Of these, 4,6% were available as immediate full OA in journals. The same study also estimated the number of articles indexed by ISI to be 945 900. In a later study looking at the article production from 2008, 1 270 000 articles were found indexed in Scopus. Of these 5,3% were in direct gold OA journals [5].

To determine the total number of articles in journals not listed in Ulrich's was not feasible within the scope of this research project; that would necessitate the creation of a journal indexing service more comprehensive than anything that exists today. Instead, subsets of DOAJ journals found in Ulrich's, Scopus, and ISI were used to estimate the shares of OA articles within those domains, matching data collected from the stratified sampling with data available from the indexes. For the total number articles in Ulrich's and ISI, figures from 2006 and 2008 were used, adjusting for annual growth of 3% [6]. For Scopus, the database could be queried directly to get the exact article count for 2009. The estimations are presented in Table 4.

**Table 4:** Estimations for 2009 shares of direct Gold OA in major indexes.

	All articles 2009	OA-articles 2009	Share of OA
Ulrich's	1 470 000	112 782	7.7%
Scopus	1 391 438	94 160	6.8%
ISI	1 033 610	61 436	5.9%

This approach has clear limitations since it was based on sampling, but the figures should still be among the most reliable published to date. Our estimates for 2009 can be compared with the estimates from the earlier studies presented above, as well as the study by McVeigh [20]. The results seem well in line, considering the growth of OA publishing over the time spanned by the studies. The share of OA articles of all peer reviewed articles, including those journals not indexed by the Ulrich's, Scopus, and ISI, would probably be even higher; however, this is only a hypothesis, and one which would be very labor-intensive to verify.

## Discussion

The results speak for the sustainability of OA as a form of scientific publishing, with a large portion of pioneer journals still active and the average number of articles per journal and year almost doubled. It can also be concluded that the relative volume of OA published peer reviewed research articles has grown at a much faster rate than the increases in total annual volume of all peer reviewed research articles. Within the last few years some high-volume and high-impact journals have made the switch to OA which further increases the relative share of openly published research.

OA journals also benefit from the fact that researchers and other potential readers increasingly use general search engines and free search engines like Google Scholar to search for articles, as their material then is on equal terms with traditional subscription articles.

Behind the aggregate numbers are a very heterogeneous lot of OA journals. The majority are relatively newly founded journals that have been open access online-only journals from the start. A notable minority also consists of older established journals, particularly journals published by scientific societies, which started making OA online-versions of their journal available in parallel to the printed subscription version. A large share of born-OA journals have been founded by individual scholars on tailor-made IT-platforms. These dominated the picture in the 1990s. Since the year 2000 a number of professionally operating specialized OA publishers have also entered the market, mainly using author-side publishing charges as form of finance and benefiting from economies of scale.

**Figure 4:**
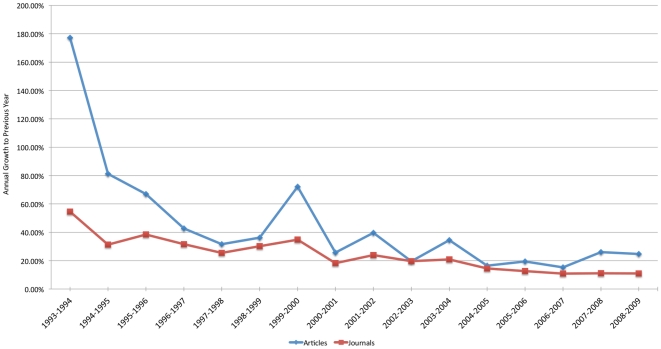
The three major phases of OA development.

The development during the years 1993–2009 can roughly be divided into three distinct phases, represented visually in Figure 4: Pioneering years (1993–1999), Innovation years (2000–2004), Consolidation years (2005–2009). It should be pointed out that despite the quantitative analysis starting at the year 1993, individual journals had already adopted OA models of publishing before that. Early examples include ‘New Horizons in Adult Education and Human Resource Development’ (1987-Present) [29] and ‘The Public-Access Computer Systems Review’ (1989–2000) [30], both of which content were distributed in plaintext through mailing lists during the early years.

During the pioneering years (1993–1999) year-to-year growth for both articles and journals was aggressive; however, one has to remember that total numbers for these years were fairly modest as presented in the results section. OA journals were almost exclusively founded by scholars or groups of scholars and published on technically simple platforms. The “business model” commonly consisted of voluntary labor combined with the possibilities of using the editor's university web servers free of cost. The model bears similarities to the traditional Open Source software production model.

During the innovation years (2000–2004) strong growth was maintained for both published OA journals and articles. New business models for running OA operations on a wider scale emerged. The author charge was pioneered by the new OA publisher BioMedCentral, which, interestingly, was funded by venture capital and was later purchased by Springer in 2008. Public Library of Science was able to establish a handful of very high-quality journals using a substantial start-up grant. In this period the general digitization of established printed journals was rapid and several society journals started to use the services of HighWire Press [31]. In other countries and regions OA publishing portals (i.e. Scielo in Latin America and J-stage in Japan) enabled the journals they serviced to get a much wider global exposure for their articles. In 2004 mainstream publishers started experimenting with the hybrid model (e.g. Springer Open Choice), which allowed authors of articles in traditional subscription journals to open up their article for a fee. It was also in this period that OA advocacy became much more visible. Several web declarations (e.g. the Budapest Open Access Initiative) laid down the central principles of OA, and conferences and even conference series dedicated to OA emerged.

During the consolidation years (2005–2009) year-to-year percentual increases for article volume has decreased from the peak years; however, growth has still been around 20% annually with publishing volume numbers dwarfing those of the earlier time periods. More and more infrastructure supporting OA publishing has emerged. The Directory of Open Access Journals (DOAJ) has become the primary index of OA journals, and also provides long term archiving possibilities via an agreement with the Dutch National Library. Increasing numbers of individual journals have, and are adopting, the free Open Journal Systems software. Licensing agreements suitable for OA journals, primarily versions of Creative Commons licenses, have also gained increasing acceptance. In the past few years several new commercial OA publishers have entered the market. Some of the big and long-established scientific publishers have started to offer open access journals funded by author charges on a small scale. The Open Access Scholarly Publishers Association has been founded to help OA publishers and to set quality standards. Authors thinking about publishing in OA journals are also helped by the fact that many research funders nowadays allow OA publishing costs to be included in research budgets, and that some universities have set aside earmarked funds for this purpose.

This study and its findings serve as a natural continuation of existing research. As outlined in the section describing earlier research, there have been several studies inquiring into the same phenomenon. However, the chosen method of sampling and data collection add precision and uniformity to elaborating on questions about the longitudinal development of OA which has not been possible before. Benefiting from having a multilingual research group, this study can be considered to be one of the most inclusive so far with regards to sampling, as no journals had to be excluded from the sample due to language issues. Furthermore, due to the use of freely available source materials, the study serves as a platform for future studies to extend upon with a larger sample or an extended observation time frame.
